# Biochemical Characterization of a Structure-Specific Resolving Enzyme from *Sulfolobus islandicus* Rod-Shaped Virus 2

**DOI:** 10.1371/journal.pone.0023668

**Published:** 2011-08-17

**Authors:** Andrew F. Gardner, Chudi Guan, William E. Jack

**Affiliations:** New England Biolabs, Inc., Ipswich, Massachusetts, United States of America; Institut Pasteur, France

## Abstract

*Sulfolobus islandicus* rod shaped virus 2 (SIRV2) infects the archaeon *Sulfolobus islandicus* at extreme temperature (70°C–80°C) and acidity (pH 3). SIRV2 encodes a Holliday junction resolving enzyme (SIRV2 Hjr) that has been proposed as a key enzyme in SIRV2 genome replication. The molecular mechanism for SIRV2 Hjr four-way junction cleavage bias, minimal requirements for four-way junction cleavage, and substrate specificity were determined. SIRV2 Hjr cleaves four-way DNA junctions with a preference for cleavage of exchange strand pairs, in contrast to host-derived resolving enzymes, suggesting fundamental differences in substrate recognition and cleavage among closely related *Sulfolobus* resolving enzymes. Unlike other viral resolving enzymes, such as T4 endonuclease VII or T7 endonuclease I, that cleave branched DNA replication intermediates, SIRV2 Hjr cleavage is specific to four-way DNA junctions and inactive on other branched DNA molecules. In addition, a specific interaction was detected between SIRV2 Hjr and the SIRV2 virion body coat protein (SIRV2gp26). Based on this observation, a model is proposed linking SIRV2 Hjr genome resolution to viral particle assembly.

## Introduction

Holliday junction resolving enzymes are structure-specific endonucleases that catalyze key steps during DNA homologous recombination and replication [1]. Resolving enzymes have been identified in all domains of life including bacteria (RuvC), archaea (Hjc), and eukarya (Human GEN1) [2]. Resolving enzymes have attracted intense interest as a model to understand the molecular basis for substrate recognition and cleavage of four-way junctions [2]. Variations in resolving enzyme sequence bias, cleavage pattern, and substrate specificity suggest that a variety of mechanisms have evolved to cleave four-way junctions [3], [4], [5], [6], [7], [8], [9], [10], [11]. *Sulfolobus islandicus* rod shaped virus 2 (SIRV2) infects the archaeon *Sulfolobus islandicus* at extreme temperature (70°C–80°C) and acidity (pH 3) and encodes a 14 kD Holliday junction resolving enzyme (SIRV2 Hjr). Hjr protein sequences are conserved among rudiviruses including *Acidianus* Rod-Shaped virus 1 (ARV1), *Stygioglobus* rod-shaped virus (SRV), and *Sulfolobus islandicus* rod-shaped viruses 1 (SIRV1) and 2 (SIRV2), and have been proposed as key enzymes in rudivirus genome replication [12]. Specifically, during the last stage of SIRV2 replication, multiple double-stranded SIRV2 genomes are catenated. At the junctions between genome monomers, opposing inverted terminal repeats can be extruded to form hairpin four-way junctions. SIRV2 Hjr is proposed to introduce symmetrical nicks across this junction to resolve the concatamers, producing monomer copies with linear hairpin ends [12]. Consistent with its proposed biological role, SIRV2 Hjr was previously shown to cleave four-way junctions *in vitro* [13], [14]. SIRV2 Hjr is related to the well-studied resolving enzymes from *Sulfolobus*, Hje and Hjc [4], [9], [15], [16], [17], [18], [19]. *Sulfolobus* Hje and Hjc are homodimeric enzymes that recognize and cleave four-way junctions by paired nicks on four-way junction arms [4], [18]. Even though a significant amount of data is available on *Sulfolobus* resolving enzymes, much less is known about the molecular mechanism of SIRV2 Hjr specificity and cleavage. Therefore, this study demonstrates a unique SIRV2 Hjr four-way junction cleavage pattern, the minimal requirements for four-way junction cleavage, and substrate specificity. Based on the biochemical analysis of SIRV2 Hjr, this study also addresses characteristics that support a role for SIRV2 Hjr in resolution following genome replication.

## Materials and Methods

### Terminology

“Hjr” will be used throughout to refer to the product of the allele from *S. islandicus* SIRV2 Hjr (or the codon-optimized allele described herein), and “MBP-Hjr” will refer to its fusion to Maltose Binding Protein below. Where necessary for clarity, the attribution will be expanded.

### Enzymes

All restriction endonucleases, modifying enzymes, DNA polymerases, nucleotides, DNA ladders, and expression vectors were from New England Biolabs. Purified *Sulfolobus solfataricus* Holliday junction endonuclease (Sso Hje) was kindly provided by Dr. Malcolm White, University of St. Andrew's, UK.

### Strains

*E. coli* strains for cloning (NEB 5 alpha) and expression (NEB Turbo and NEB T7 Express) were from New England Biolabs.

### MBP-SIRV2 Hjr gene synthesis, cloning and purification

To improve protein expression, a synthetic Hjr gene was codon optimized to reflect the codon usage of *E. coli* rather than the native *S. islandicus*. Hjr gene was synthesized by PCR amplification of overlapping oligonucleotides [20].

To assemble a template for Hjr gene synthesis, an equimolar amount (1 µM) of each overlapping oligonucleotide (Table S1) was combined in 1× Standard Taq Buffer (10 mM TrisHCl, pH 8.3, 50 mM KCl, 1.5 mM MgCl_2_) and then serially diluted by two-fold. PCR reactions (50 µL) were assembled as follows: 1× Phusion Master Mix (containing dNTPs, HF reaction buffer, and Phusion DNA polymerase), 0.5 µM Forward Primer (Table S1, primer 1), 0.5 µM Reverse Primer (Table S1, primer 10), and Hjr gene synthesis oligonucleotide template mixtures. Reactions were cycled in a PCR instrument (98°C 2 minutes followed by 25 cycles of 98°C 10 seconds, 65°C 15 seconds, 72°C for 30 seconds, followed by a final extension step at 72°C for 30 seconds). A band corresponding to the Hjr gene (405 bp) was gel purified. The Hjr codon optimized PCR product was cloned into expression vector pMAL-c4X (New England Biolabs) digested with XmnI to create a construct (pEPD) encoding an N-terminal Maltose Binding Protein (MBP) – SIRV2 Hjr fusion protein. The sequence of plasmid pEPD was verified by DNA sequencing.

For MBP-Hjr expression and purification, NEB Turbo *E. coli* was transformed with plasmid pEPD. A 1 liter NEB Turbo *E. coli*/pEPD culture was grown at 37°C to mid-log phase (OD_600_ = 0.5), whereupon protein expression was induced by addition of 0.4 mM IPTG. Cells were then incubated at 37°C for five hours, and were collected by centrifugation. The cell pellet was suspended in 0.2 L Buffer A (20 mM TrisHCl, pH 7.5, 0.2 M NaCl, 1 mM EDTA) and lysed by sonication. Cell debris was removed by centrifugation and the supernatant applied to a 15 mL amylose column. The column was washed with 0.15 L Buffer A. MBP-SIRV2 Hjr was eluted with 30 mL Buffer A containing 10 mM maltose. MBP-Hjr purification was monitored by 4–20% SDS-PAGE analysis. Fractions containing MBP-Hjr were pooled, dialysed against storage buffer (0.1 M KCl, 10 mM Tris-HCl, pH 7.4 @ 25°C, 1 mM dithiothreitol, 0.1 mM EDTA, 50% Glycerol) and stored at −20°C. A portion of MBP-Hjr was proteolysed by Factor Xa protein to separate the MBP binding domain from SIRV2 Hjr and not further purified. Activity at 55°C was comparable between the intact and proteolyzed proteins, so the MBP-Hjr fusion was used in most experiments. However, it should be noted that even though no apparent differences in overall activity were observed using the MBP-Hjr fusion, steps in the reaction pathway may be influenced by the presence of the MBP-fusion.

### MBP-*Sulfolobus islandicus* Holliday junction endonuclease (Sis Hje) gene synthesis, cloning and purification

The gene encoding a Holliday junction endonuclease from *Sulfolobus islandicus* strain Y.N.15.51 (Sis Hje) [21] was synthesized using the methods described above using the overlapping oligonucleotides presented in Table S1. Sis Hje *E. coli* codon optimized PCR product was cloned into expression vector pMAL-c4X digested with XmnI to create a construct encoding an N-terminal Maltose Binding Protein (MBP) – Sis Hje fusion protein.

### SIRV2gp26 gene synthesis, cloning and purification

A gene encoding the SIRV2gp26 coat protein was synthesized using the methods described above, with the overlapping oligonucleotides listed in Table S1. The SIRV2gp26 *E. coli* codon optimized PCR product was cloned into expression vector pMAL-c4X digested with XmnI to create a construct encoding an N-terminal Maltose Binding Protein (MBP) – SIRV2gp26 fusion protein. A portion of the MBP-SIRV2gp26 was treated with Factor Xa protease to separate MBP and SIRV2gp26 and heated to 65°C for 20 minutes to inactive the protease.

### DNA substrates

Two plasmids containing hairpin four-way junctions were constructed to assay resolving enzyme activity. pUC(AT) is a derivative of pUC19 containing an inverted repeat of twenty A and T dinucleotides ((AT)_20_) between the EcoRI and PstI sites that forms a hairpin four-way junction upon supercoiling [22], [23] (Figure S1A). Plasmid pEMM2 is derived from pNEB206A (NEB, Ipswich, MA) and contains an insert corresponding to the expected four-way junction formed between SIRV2 genome dimers during genome replication (Figure S1B). pEMM2 was constructed by annealing overlapping SIRV2 four-way junction oligonucleotides in 1× Standard Taq Buffer (10 mM TrisHCl, pH 8.3, 50 mM KCl, 1.5 mM MgCl_2_). This oligonucleotide cassette with 3′ overhangs was ligated to complementary ends on pNEB206A vector linearized by XbaI and Nt. BbvCI (New England Biolabs) to create pEMM2. The correct pEMM2 sequence was confirmed by DNA sequencing.

Synthetic oligonucleotide substrates were also used to characterize resolving enzyme specificity and activity. Four-way Junction 3 (J3) was constructed by annealing strands (25 µM each) b, h, r, and x in 1× Standard Taq Buffer. In addition, fluorescently labeled J3 four-way junctions were prepared by annealing one 6-carboxyfluorescein (FAM)-labeled strand and three unlabeled strands. A SIRV2 four-way junction was constructed by annealing strands (25 µM each) 1, 2, 3, and 4.

**Figure 1:**
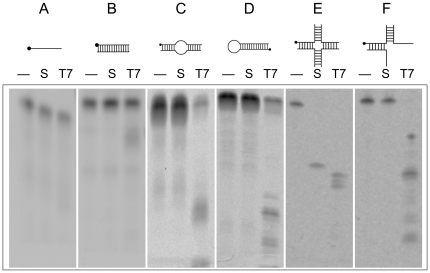
SIRV2 Hjr cleavage is specific to four-way junction DNA. Substrates (A: single-stranded DNA, B: double-stranded DNA, C: heteroduplex double-stranded DNA, D: hairpin DNA, E: four-way junction J3, F: three-way Holliday-like junction) were constructed as described in Materials & Methods and 5′ labeled with fluorescence for detection (as indicated by a black circle). Distilled water (-), Hjr (S) (20 nM) or T7 endonuclease I (T7) (7.5 nM) was incubated with 100 nM substrate in 1× ThermoPol Buffer at 55°C for 30 minutes. Reaction products were separated by 20% denaturing PAGE and fluorescence detected by a GE Typhoon scanner.

In addition to four-way junction DNA, Hjr activity was assayed on alternate DNA structures, including single- and double-stranded DNA, bulged DNA, hairpin, and three-strand Holliday-like junctions. Schematics representing the DNA structures used in these experiments are depicted in Figure 1. Oligonucleotides for DNA substrates are listed in Table S2. Double-stranded DNA was formed by annealing FAM-labeled top strand to an unlabeled complement in 1× Standard Taq Buffer. A FAM-labeled hairpin DNA mimicking the SIRV2 genome end was formed by self-annealing. Heteroduplex bulged DNA (25 µM stock) was constructed by annealing two oligonucleotides in 1× Standard Taq Buffer to create an unpaired central region flanked by complementary base pairing. A three-strand Holliday-like junction was prepared by annealing four-way junction J3 strands (FAM)-b, h, r.

### Assays for resolving enzyme activity

Four-way junction resolution was monitored by cleavage of either four-way junction containing plasmids (pUC(AT) or pEMM2) or fluorescently labeled synthetic four-way junctions. Typically in a 50 µL reaction, plasmids (11 nM) were incubated with 20 nM resolving enzyme in 1× ThermoPol Buffer (20 mM Tris-HCl, 10 mM (NH_4_)_2_SO_4_, 10 mM KCl, 2 mM MgSO_4_, 0.1% Triton X-100, pH 8.8 @ 25°C) at 55°C for one hour. Reaction products were separated by agarose gel electrophoresis. Synthetic four-way junctions were constructed as described above and one strand was fluorescently labeled on its 5′ end. In a 20 µL reaction, a synthetic four-way junction (J3 or SIRV2 at 100 nM) was incubated with 20 nM resolving enzyme in 1× ThermoPol Buffer (unless otherwise noted) at 55°C for one hour. FAM-labeled 16-mer and 18-mer were size standards during electrophoresis (Table S2). Reaction products were separated by 20% denaturing PAGE and quantified using the fluorescence detected by a GE Typhoon scanner.

### Requirements for SIRV2 Hjr cleavage

Hjr cleavage activity was assayed with synthetic DNA four-way junction J3 under a variety of reaction conditions to determine requirements for cleavage. Four-way junction J3 (FAM-labeled on strand b) (100 nM) and MBP-Hjr (20 nM) were incubated in reaction buffer at 55°C for 30 minutes. Reaction buffers varied in pH (4–10), divalent cation (MgCl_2_, MnCl_2_, ZnSO_4_, CaCl_2_, CoCl_2_), or concentrations of NaCl (0–500 mM), NH_4_SO_4_ (0–200 mM), or MgCl_2_ (0–100 mM). Sodium acetate (10 mM) was used in the pH range of 4.0–6.0 and TrisHCl (10 mM) was used in the pH range of 7.0–10. Reactions were halted by addition of 50% formamide and 5 mM EDTA. Reaction products were separated by 20% denaturing PAGE and fluorescence detected on a GE Typhoon scanner.

### Hjr substrate specificity

To determine Hjr substrate specificity, a panel of DNA substrates including single- and double-stranded DNA, bulged DNA, hairpin, and three-strand Holliday-like junctions were prepared as described above. MBP-Hjr (20 nM) or T7 endonuclease I (7.5 nM) (New England Biolabs) was incubated with 0.10 µM DNA in 1× ThermoPol Buffer at 55°C for 30 minutes. Reaction products were separated by 20% denaturing PAGE and quantified using the fluorescence detected by a GE Typhoon scanner.

### Characterization of Hjr four-way junction cleavage products

In a 0.10 mL reaction, pUC(AT) (25 nM) was incubated with 20 nM MBP-Hjr in 1× ThermoPol Buffer at 55°C. Linearized products were gel purified by QiaPrep PCR purification kit (Qiagen) and eluted in 0.10 mL of EB buffer. Linearized products (50 µL) were treated with 400 U T4 DNA ligase in 1× T4 DNA ligase buffer for one hour at room temperature to seal hairpin nicks. Lambda exonuclease treatment was carried out in a 30 µL reaction by mixing 3 µL 10× lambda exonuclease buffer, 5 U lambda exonuclease and 26 µL of linearized products, and incubated at 37°C for one hour to degrade DNA with free 5′ termini. Reaction products were separated by agarose electrophoresis.

### Immunoprecipitation

Protein extracts of SIRV2-infected *S. islandicus* were prepared from 0.25–0.5 L cultures after concentration of the cells by centrifugation, suspension of the cells in 25 mL of 150 mM NaCl, 20 mM TrisHCl, pH 7.5, and 1 mM EDTA and lysis by sonication. Cell debris was removed by centrifugation and the supernatant centrifuged a second time to further remove cell debris. The resulting clarified cell-free lysate was used for further studies. In a 0.5 mL reaction, 10 µg MBP-Hjr and incubated with ∼1 mg SIRV2-infected *S. islandicus* extract at 4°C for 16 hours with shaking in NEBuffer 3 (100 mM NaCl, 50 mM Tris-HCl, 10 mM MgCl_2_, 1 mM dithiothreitol, pH 7.9 @ 25°C). Anti-MBP magnetic beads were added and affinity complexes were magnetically separated and washed five times with 1.0 mL of NEBuffer 3 to elute non-specifically bound protein. The remaining specific protein complexes were eluted by boiling in 1× SDS-PAGE loading buffer for 5 minutes and analyzed by SDS-PAGE.

### Identification of interacting proteins by Mass Spectrometry

Proteins eluted from MBP-Hjr capture experiments were digested into peptides with trypsin and run on an LC/MS-MS for peptide analysis at the New England Biolabs Proteomic Facility. Peptide masses were compared to a database of *S. islandicus* strain Y.N.15.51 (accession: NC_012623 [21]) and SIRV2 protein sequences using Spectrum Mill software (Agilent Technologies). Peptides that did not exactly match the amino acid sequences in the database, had alternatively charged states, or were modified by phosphorylation or glycosylation were not scored as positives. The sequence of host *S. islandicus* LAL 14/1 is not publicly available, forcing the use of data from closely related strains, so the list of identified proteins is expected to be an underestimate of true positive interactors in the actual extract. Data was filtered for quality with an MS/MS Score cut off of 15. Proteins from *S. islandicus* or SIRV2 were identified by sequence comparison using a BLAST [24] similarity score cut off of E<0.1. Interacting proteins identified by mass spectrometry were tabulated and grouped according to their presumed functional role.

### Interaction between Hjr and SIRV2gp26 coat protein

To directly test the interaction between Hjr and SIRV2gp26 coat protein *in vitro*, anti-MBP::MBP-Hjr magnetic beads (hereafter called MBP-Hjr affinity beads) were prepared by mixing 10 µg MBP-Hjr with 0.1 mg anti-MBP magnetic beads pre-equilibrated in NEBuffer 3 (New England Biolabs). Bead complexes were mixed thoroughly and incubated at 4°C with shaking for 1 hour. A magnet was applied and supernatant was decanted and bead complexes were washed 5 times with 1× NEBuffer 3. These MBP-Hjr affinity beads (10 µg) were incubated with approximately 10 µg SIRV2gp26 prepared as described earlier at 25°C for 2 hours with shaking in 0.5 mL 1× NEBuffer 3. Affinity complexes were separated by a magnet and washed five times with 1.0 mL of NEBuffer 3 to remove any non-specifically bound protein. Protein complexes were eluted by boiling in 1× SDS-PAGE loading buffer for 5 minutes and analyzed by SDS-PAGE. Background binding was assessed using MBP affinity beads prepared with unfused MBP (anti-MBP::MBP5).

## Results

### SIRV2 Hjr cleaves four-way junction but not branched DNA

Previously described viral resolving enzymes cleave a variety of branched DNA structures formed during replication [11], [25], [26]. To test if Hjr substrate requirements parallel those of other viral resolving enzymes, Hjr and bacteriophage T7 endonuclease I activities on the panel of DNA substrates depicted in Figure 1 were compared. In contrast to T7 endonuclease I, Hjr only cleaves four-way junction DNA and is inactive on single- or double-stranded DNA, hairpin DNA, heteroduplex loops (bulges), and three-way Holliday-like junctions (Figure 1). *S. solfataricus* Hje shows a similarly narrow substrate range and only cleaves four-way junction DNA structures [9].

### Hjr activity on four-way junction DNA

**Figure 2:**
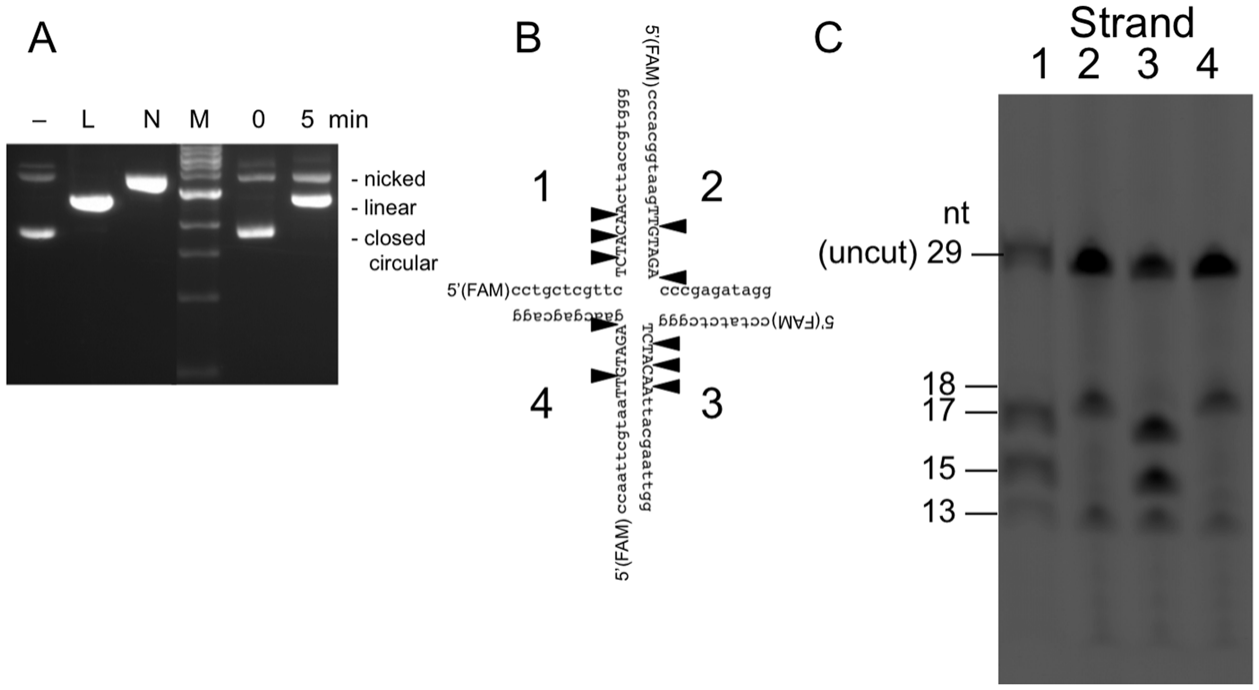
SIRV2 Hjr cleaves four-way DNA junctions. (A) Cleavage of plasmid pUC(AT) by Hjr was monitored over time by agarose gel electrophoresis. The mobility of nicked pUC(AT) was established by treating the plasmid with the nicking enzyme Nt. BstNBI (Lane N), and that of the linear form by digestion with HindIII (Lane L). The NEB 1 kb DNA ladder (M) was used as a reference. (B) A four-way junction sequence and structure is shown with uppercase nucleotides correspond to native SIRV2 sequence. Strands are designated 1–4. SIRV2 Hjr cleavage sites are noted by triangles. (C) A four-way junction DNA substrate corresponding to the SIRV2 concatamer junction sequence (shown in B) was constructed by annealing four oligonucleotides, three unlabeled and one FAM-labeled; differently-labeled substrates are designated 1, 2, 3, or 4. MBP-Hjr was incubated with these (Lanes 1–4 respectively) at 55°C for 1 hour in 1× ThermoPol Buffer. Reaction products were separated by denaturing 20% PAGE and quantified using a phosphoimager. Fragment sizes are indicated.

To identify DNA structural elements and sequences required for Hjr cleavage, activity on a variety of four-way junction DNAs was examined. First, Hjr was tested on plasmid substrates containing junctions that resemble hairpin four-way junction substrates formed during SIRV2 replication *in vivo*. Plasmid pUC(AT) contains an AT-rich hairpin four-way junction and has been used as a substrate to study T7 endonuclease I and fowlpox resolving enzyme (Figure S1A) [23], [27]. Plasmid pEMM2 includes a hairpin four-way junction that mimics the sequence formed at the SIRV2 concatamer junctions during replication (Figure S1B). Hjr cleaves the pUC(AT) four-way junction to convert closed circular pUC(AT) to a linear form (Figure 2A). Hjr also cleaves the four-way junction structure in plasmid pEMM2 to a linear form (data not shown). The Hjr-cleavage site was mapped by restriction fragment length analysis using XmnI and HindIII and shown to be specific for the DNA four-way junction (Figure S2). Relaxed nicked and linear pUC(AT) and pEMM2 are not substrates, presumably because the four-way junctions do not form in the absence of supercoiling (data not shown). In addition, plasmids lacking four-way junctions are also not substrates (data not shown).

Hjr cleavage was then tested on a synthetic four-way junction designed to mimic possible structures formed during SIRV2 replication. The SIRV2 cruciform sequence allows four-way junction motion along the duplex and as the four-way junction migrates along the substrate, the crossover points may vary. The Hjr dimer recognizes four-way junction structures and makes a nick on pairs of four-way junction strands [2]. Hjr nicks strands 1 and 3 (Lanes 1, 3) at three paired sites and makes two paired nicks on the strands 2 and 4 of SIRV2 four-way junction DNA (Lanes 2, 4) (Figure 2B, C). The observed multiple cleavage sites could reflect different four-way junction configurations due to migration, each cleaved at a fixed distance from a crossover point but at different positions relative to the end or alternatively multiple cleavage sites at a single crossover point.

**Figure 3:**
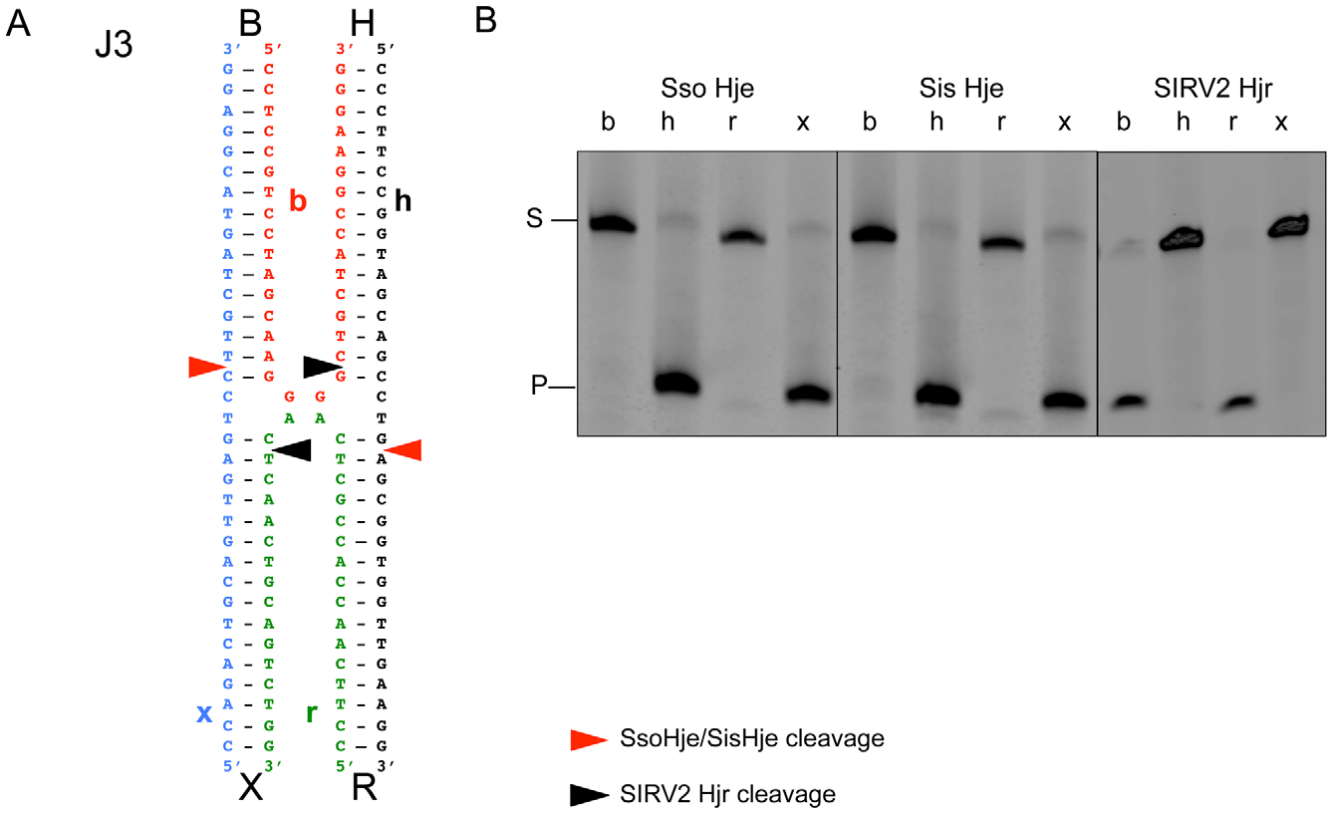
*S. solfataricus* and *S. islandicus* Hje and SIRV2 Hjr cleaves of four-way junction J3 with opposite polarity. (A) Resolving enzyme cleavage sites are shown on a schematic representation of four-way junction J3 with continuous strands colored blue (x) and black (h) and exchange strands colored red (b) and green (r). Sso Hje and Sis Hje cleave four-way junction J3 junction two base pairs 3′ from the junction in a symmetrical fashion on the h and x continuous strands while Hjr cleaves two base pairs 3′ from the junction in a symmetrical fashion on the b and r exchange strands. (B) Four-way junction J3 DNA (100 nM) 5′ FAM-labeled either on the h, b, r, x strand was incubated with 20 nM *S. solfataricus* (Sso Hje), *S. islandicus* Hje (Sis Hje), or Hjr in 1× ThermoPol Buffer at 55°C for 60 minutes. Reaction products were separated by denaturing 20% PAGE. S and P represent substrate (34-mer) and product (18-mer) bands, respectively.

After initial characterization of Hjr activity with the mobile SIRV2 four-way junction, an alternate well-characterized fixed four-way junction DNA substrate (Junction 3 (J3)) was used for further detailed characterization. By utilizing the well-defined junction J3 as a substrate for Hjr cleavage, comparisons can be made to other resolving enzyme cleavage patterns described in the literature that use the same J3 substrate [9], [28], [29]. J3 is composed of four hybridized DNA strands: two DNA strands are continuously stacked and the other two strands base pair with one continuous strand then switch to base pair with the other continuous strand on the adjacent helix (Figure 3A) [30]. The pair of DNA strands that maintain base stacking through the junction are referred to as the continuous strands (strands h and x) while strands that exchange between helices are the exchange strands (strands b and r) (Figure 3A), and bases at the junction are stacked with different strands on the two faces [31]. Local sequence properties change the probability of adopting the stacking switch. The four-way junction J3 favors an isoform conformation with strands h and x as continuous strands and strands b and r as exchange strands [31].

Four-way junction J3 was fluorescently labeled on either the b, h, r, or x strand. Both Sso and Sis Hje cleave preferentially on the continuous strands (h, x) of the J3 four-way junction (Figure 3B). In contrast, Hjr preferentially cleaves the exchange strands (b, r) of this same four-way junction (Figure 3B).

### Requirements for SIRV2 Hjr four-way junction cleavage

The reaction conditions for Hjr cleavage were investigated in detail using the synthetic J3 four-way junction. Despite the acidic growth environment, the internal pH of *Sulfolobus islandicus* is neutral. The pH optimum for Hjr activity reflects its native cytosolic environment with optimal activity between pH 7 to 9, partial activity at pH 6 and 10 (80% or 70% activity, respectively) and no activity below pH 5 (Figure S3A). Consistent with previous studies, a divalent cation (MgCl_2_ or MnCl_2_) is required for resolving enzyme activity (Figure S3C) [28]. Hjr activity is optimal between 0.5 and 20 mM MgCl_2_ (Figure S3B). Hjr is minimally active with CaCl_2_ and not active with cofactors ZnSO_4_ or CoCl_2_ (Figure S3C). NaCl or (NH_4_)_2_SO_4_ are not required for Hjr activity and do not stimulate cleavage (Figure S3D, E). However, Hjr activity is inhibited by higher concentrations of NaCl (>250 mM) and (NH_4_)_2_SO_4_ (>10 mM) (Figure S3D, E).

### SIRV2 Hjr four-way hairpin junction cleavage results in linear DNA with hairpin termini

**Figure 4:**
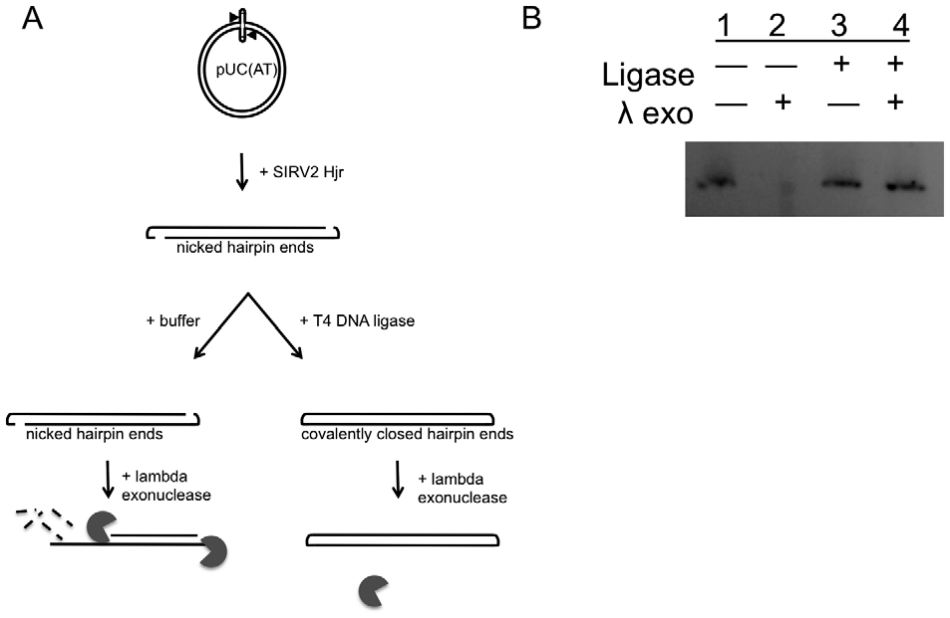
SIRV2 Hjr cleaves hairpin four-way junctions to produce linear fragments with nicked hairpin ends. (A) Hjr was used to cleave the hairpin four-way junction containing plasmid, pUC(AT), to generate linear fragments with nicked hairpin ends. The linear fragments were then incubated with either buffer alone or T4 DNA ligase to seal nicks. Lambda exonuclease (gray shape) was then added to degrade DNA having free 5′ ends (dotted lines). (B) Hjr cleaved pUC(AT) products were incubated with buffer alone (-) (Lane 1), with lambda exonuclease (Lane 2), with T4 DNA ligase (Lane 3) or with T4 DNA ligase then lambda exonuclease (Lane 4) and separated by agarose gel electrophoresis.

The last stage of SIRV2 replication is envisioned as a concatamer joined by a hairpin four-way junction. Hjr is proposed to introduce symmetrical nicks across this junction to resolve the concatamers, producing single molecules with linear hairpin ends [12]. To test this model, a plasmid-encoded hairpin four-way junction was cut with SIRV2 and the nature of the ends was determined as schematically depicted in Figure 4A. First, the four-way junction containing plasmid, pUC(AT), was cleaved with Hjr to generate a linear fragment and then incubated with buffer alone or with T4 DNA ligase (Figure 4A). Lambda exonuclease was added to digest DNA with free 5′ ends. As predicted, linear fragments are sensitive to lambda exonuclease digestion in the absence of ligation due to free 5′ ends (Figure 4B). However, linear fragments treated with T4 DNA ligase are resistant to lambda exonuclease cleavage suggesting that ends are nicked hairpins that can be ligated to form contiguous covalently closed linear DNA. Therefore, these data support a model in which Hjr cleaves genome concatamers at hairpin four-way junctions to produce single genome copies having nicked hairpin ends that can then be sealed by DNA ligase to form covalently closed molecules.

### SIRV2 Hjr interacts with host DNA binding proteins and SIRV2 coat protein (gp26)

To investigate what protein partners might function with Hjr, immunoprecipitation was used. MBP-Hjr was incubated with a cell-free extract of SIRV2-infected *S. islandicus*, containing protein, lipid, and nucleic acids. Components bound to the resolving enzyme were captured by magnetic anti-MBP beads and washed to reduce non-specific binding. Treatment of the washed beads with Factor X protease released Hjr and bound proteins. Several proteins in the 10–20 kD range and the 40–50 kD range were observed by SDS-PAGE (data not shown).

Proteins captured by MBP-Hjr immunoprecipitation were identified by mass spectrometry and are listed according to their presumed functional role (Table S3). MBP-Hjr forms complexes with proteins including the DNA binding proteins, Sso10b, Sso7d, Cren7 and Alba (Table S3). Interactions between MBP-Hjr and these DNA binding proteins could be a result of direct protein:protein interactions or may result from binding between MBP-Hjr and a nucleic acid that is in turn bound by a DNA binding protein. Most strikingly, an interaction between Hjr and the coat protein, SIRV2gp26 was identified with a high confidence value (Table 1). Further studies using purified proteins were implemented to verify this interaction with the SIRV2 coat protein.

**Table 1:** MS/MS data for SIRV2gp26 coat protein captured by SIRV2 Hjr immunoprecipitation.

Spectra (#)^a^	Distinct Peptides^b^	MS/MS Score^c^	% AA coverage^d^	Accession (#)^e^	Annotation^f^
4	1	20	22	SIRV2gp26	SIRV2gp26 coat protein

#	Score	Fwd-Rev Score	Spectrum Intensity	Sequence^g^
1	20.69	20.69	4.88e+007	(K)FNAFSNPTVASTILSNVSPVAQQNFQTNVPK(F)
2	19.56	19.56	1.70e+007	(K)FNAFSNPTVASTILSNVSPVAQQNFQTNVPK(F)
3	18.71	18.71	4.79e+007	(K)FNAFSNPTVASTILSNVSPVAQQNFQTNVPK(F)
4	14.81	14.81	1.66e+007	(K)FNAFSNPTVASTILSNVSPVAQQNFQTNVPK(F)
>SIRV2gp26 h MAKGHTSRSYSQRYAKWQA**KFNAFSNPTVASTILSNVSPVAQQNFQTNVPKF**TSVNENVSAVLTQYGITGPNRAIYQGFGLKVARALNRIGSGPALVNMINGLKGYYISAFNANPQVLDAVVNIITGSPTGYVS				

a A single MS spectral scan of a defined mass range may contain many distinct peaks, each with a characteristic mass/charge ratio – m/z. MS/MS spectra are the total number of peptide spectra generated by MS/MS from a single distinct peak observed in a MS spectrum which match the predicted *in silico* fragments from a translated database of the SIRV2 genome (accession number: NC_004086 [12]).

b Total number of unique spectra detected and assigned to one peptide.

c Spectra were scored by Spectrum Mill and assigned a quality score based on peak match and quality. Matching spectra were associated with a peptide sequence. A total MS/MS score was generated by summing only the highest scoring MS/MS spectrum for each peptide.

d Percent of identified peptide amino acids matching total protein amino acids sequence.

e Protein accession number from translated SIRV2 genome; accession number: NC_004086 [12]).

f Proteins were annotated by BLAST similarity (e<0.1) [24].

g SIRV2gp26 peptide identified by mass spectrometry.

h SIRV2gp26 amino acid sequence highlighting putative trypsin digestion sites (underlined) and peptide identified by mass spectrometry (bold).

### SIRV2 Hjr and SIRV2 coat protein (gp26) interact *in vitro*

**Figure 5:**
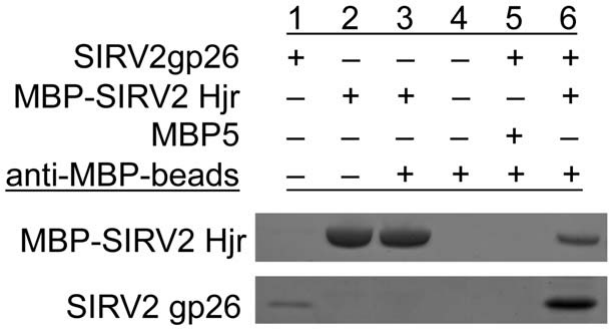
SIRV2 Hjr interacts with SIRV2gp26 coat protein *in vitro*. The interaction of MBP-Hjr and SIRV2gp26 coat protein was confirmed by immunoprecipitation. Protein complexes were eluted and analyzed by SDS-PAGE stained with Coomasie Blue dye: Lane 1: SIRV2gp26. Lane 2: MBP-Hjr. Lane 3: anti-MBP beads:MBP-Hjr. Lane 4: anti-MBP beads. Lane 5: anti-MBP beads:MBP5+SIRV2gp26. Lane 6: anti-MBP beads:MBP-Hjr+SIRV2gp26.

The putative direct interaction between purified MBP-Hjr and SIRV2gp26 was tested *in vitro*. As detected by SDS-PAGE, magnetic anti-MBP::MBP-Hjr beads pulled down SIRV2gp26 (Figure 5). Control reactions with MBP5 alone did not pull down SIRV2gp26 suggesting that the interaction is specific for Hjr rather than for the MBP domain of the fusion protein.

## Discussion

To address the molecular basis for substrate recognition and cleavage, we characterized the resolving enzyme from the hyperthermophilic virus, SIRV2. Hjr cleaves four-way junctions without cleaving other related DNA structures and contrasts with the bacteriophage resolving enzymes T4 endonuclease VII and T7 endonuclease I that recognize a wide range of DNA structures including four-way junctions, Y-structures, heteroduplex loops, single-strand overhangs, nicks, gaps, apyrimidinic sites, and base mismatches [32], [33], [34]. T4 endonuclease VII and T7 endonuclease I have large electropositive areas on the protein surface that bind to the four-way junction DNA backbone over a large area and are flexible and broad enough to allow binding and cleavage a variety of branched DNA substrates [35], [36]. Unlike bacteriophage resolving enzymes, Sso Hje, Sso Hjc, and SIRV2 Hjr substrate specificity is narrow and limited to X-shaped four-way junctions. The Sso Hje three-dimensional structure provides a model to explore elements that determine the narrow substrate range of Sso Hje and SIRV2 Hjr. Electropositive patches are arranged on the Sso Hje and Sso Hjc DNA binding surface in an X-shaped pattern [4], [18]. Presumably, this pattern of electropositive residues favors binding of X-stacked four-way junctions by Sso Hje and Hjc (and by extension, SIRV2 Hjr). Further structural studies of SIRV2 Hjr in complex with DNA will further reveal if positively charged surface amino acids are arranged in a pattern to bind X-stacked four-way junction substrates to confer substrate specificity.

**Figure 6:**
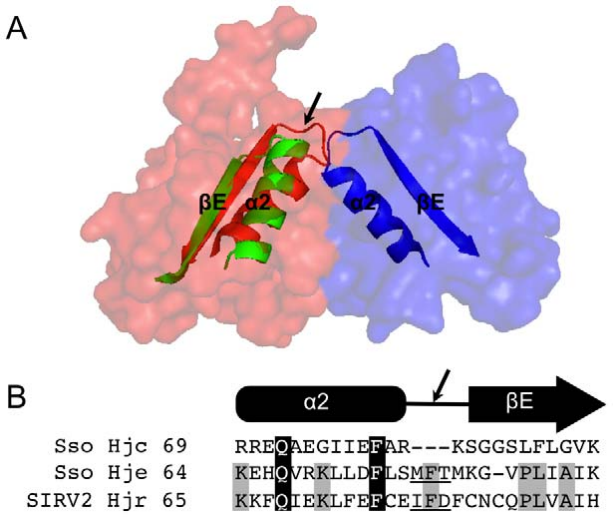
Structural variation at the Sso Hje and Hjc dimer interface modulates cleavage specificity. (A) Sso Hje (accession: 1OB8) and Sso Hjc (accession: 1HH1) three-dimensional structures were aligned using MacPymol. Sso Hje monomers (red and blue) are visualized by a surface view to highlight the dimer arrangement. A domain formed by helix alpha-2 and beta strand beta-E form part of the dimer interface (arrow). In insertion in the loop between helix alpha-2 and beta strand beta-E (arrow) (Sso Hje: red and blue; Sso Hjc: green) shifts dimer conformation and may confer strand cleavage specificity. (B). The amino acid sequence of the Sso Hje, Sso Hjc, and SIRV2 Hjr dimerization interface helix alpha-2 and beta strand beta-E were aligned by Clustal W and the insertion loop is underlined. Amino acids conserved between Sso Hje, Sso Hjc, and SIRV2 Hjr are shaded in black and those conserved between Sso Hje and SIRV2 Hjrare shaded in gray.

SIRV2 Hjr has a unique strand cleavage preference that may reflect fundamental differences in Holliday junction recognition and cleavage. This preference is observed even while sharing sequence similarity with Sso Hje and Sso Hjc. Sso Hje is specific for cleavage of continuous strands in four-way junction J3 while Sso Hjc cleaves both continuous and exchange strands. SIRV2 Hjr presents a third cleavage pattern by nicking exchange strand pairs. Previous studies of Sso Hje and Hjc have suggested a structural basis to account for differences in resolving enzyme specificity and cleavage patterns (1). Sso Hje and Sso Hjc are both homodimers stabilized along the dimer interface by interactions between monomer amino acids (Figure 6A). Even thought the dimer interface is distal to the DNA binding region, Middleton and coworkers have argued that positioning of the dimers influences positioning of adjacent catalytic residues on the DNA. When Sso Hje and Sso Hjc three-dimensional structures are superimposed, the main chain Cα positioning is structurally well conserved with the largest differences observed at the dimer interface (Figure 6A). Specifically, an insertion of three large hydrophobic residues (M77, F78, M80) in a loop between helix α2 and beta strand βE of each Sso Hje monomer shifts the dimer conformation and may position catalytic residues for cleavage of continuous strands of a four-way junction. In contrast to Sso Hje, a relatively short Sso Hjc loop connecting helix α2 and beta strand βE may confer additional flexibility to allow cleavage of both exchange and continuous strands in a four-way junction. SIRV2 Hjr, like Sso Hje, also contains an insertion in the equivalent loop although the sequences diverge. Most notably, SIRV2 Hjr contains three cysteines (C77, C82, C84) in the insertion loop. These cysteines may form intra- or inter-monomer disulfide bonds to alter resolving enzyme conformation and thereby position adjacent catalytic residues to favor cleavage of exchange rather than continuous strands (Figure 6B). Therefore, variations in resolving enzyme dimer interfaces may uniquely position catalytic residues for pairwise nicking on either the continuous (Sso Hje), exchange (SIRV2 Hjr), or both (Sso Hjc).

The SIRV2 Hjr substrate cleavage specificity has the characteristics to function as a replication resolving enzyme in a poxvirus-like mode of viral DNA replication. In such a mechanism, linked genome concatamers are resolved via cleavage during replication [12]. SIRV2 Hjr action on such hairpin four-way junction concatamer DNA would produce linear DNA products with nicked hairpin termini, which in turn could serve as nicked templates for further replication. Therefore, the SIRV2 resolving enzyme likely plays a central role in the final stages of SIRV2 replication and functions in the general mechanism for separating concatamers during replication of linear viral genomes.

Finally, we have shown that in addition to cleaving four-way junctions, Hjr may have a role in viral assembly. It is tempting to speculate that an interaction between Hjr and coat protein could be the signal for assembly of the coat protein into a superfilament that surrounds the resolved SIRV2 genome, thereby forming the virion body. Known signals for viral packaging include DNA sequences, RNA structures, and non-structural proteins that recruit viral structural proteins to initiate assembly [37], [38], [39], [40], [41], [42], [43], [44], [45], [46], [47], [48], [49]. For instance, vaccinia virus nucleates virion assembly via a transient interaction between a hairpin terminus-binding protein, I6, and the vaccinia virus coat protein [48], [49]. Therefore, like vaccinia virus I6 protein, the interaction between Hjr and coat protein could ensure that resolved, single genome copies rather than concatamers are packaged into virus particles. In addition, because both Hjr and coat protein sequences are highly conserved among rudiviruses, this nucleation model could also serve as a general strategy for virion packaging in related rudiviruses, *Acidianus* Rod-Shaped virus 1 (ARV1), *Stygioglobus* rod-shaped virus (SRV), and *Sulfolobus islandicus* rod-shaped virus 1 (SIRV1). Given the similarity between archaea and eukarya, it is also conceivable that similar encapsidation mechanisms may also exist in eukaryotic viruses.

## Supporting Information

Figure S1 **Four-way junction DNA structures and sequences.** (A) A poly(AT)_20_ cassette in pUC(AT) can form a four-way junction structure. (B) pEMM2 contains a four-way junction sequence at the junction of SIRV2 concatamers. These figures represents one of many conformations of possible mobile four-way junction structures. The four-way junction center may shift from what is represented. Gray regions are vector sequence.
(PDF)

Figure S2 **Mapping resolvase cleavage site on plasmid pEMM2.** Plasmid pEMM2 contains a four-way junction sequence mimicking the four-way junction formed at SIRV2 concatamer junctions. pEMM2 (100 nM) was incubated with T7 endonuclease I (7.5 nM) (Lane 2), MBP-Hjr (20 nM) (Lane 3), or MBP-Hjr (2 nM) (Lane 4) at 37°C (T7 endonuclease I) or 55°C (MBP-Hjr) for 30 minutes. Then reaction products were then digested with 10 Units of XmnI at 37°C for one hour and separated by agarose gel electrophoresis. As a control (Lane 1), 1 µg pEMM2 was digested with 10 Units of XmnI and HindIII (proximal to the four-way junction) to generate a 921 bp and 1851 bp fragment. pEMM2 was digested with XmnI alone (Lane 5), XmnI and HindIII (proximal to the four-way junction). Plasmid not cleaved by T7 endonuclease I or SIRV2 Hjr and then cleaved with XmnI will generate a linear 2772 bp band. As expected, a double stranded break generated from T7 endonuclease I and MBP•SIRV2 Hjr cleavage mapped to the cruciform region of pEMM2 to generate a ∼875 bp and 1897 bp fragment (Lanes 2, 3, 4). (B) A plasmid map of pEMM2 illustrating HindIII, XmnI and cruciform cleavage site.
(PDF)

Figure S3 **Requirements for SIRV2 Hjr four-way junction cleavage.** Hjr cleavage activity was assayed with DNA four-way junction J3 under a variety of reaction conditions to determine requirements for cleavage. Four-way junction J3 (FAM-labeled on strand b) (100 nM) and Hjr (20 nM) were incubated in reaction buffer at 55°C for 30 minutes. Reaction buffers varied pH (4–10), divalent cation (MgCl_2_, MnCl_2_, ZnSO_4_, CaCl_2_, CoCl_2_), concentration of NaCl (0–500 mM), NH_4_SO_4_ (0–200 mM), or MgCl_2_ (0–100 mM). Reactions were quenched by addition of 50% formamide and 5 mM EDTA. Reaction products were separated by 20% denaturing PAGE and fluorescence quantified using a GE Typhoon scanner. S and P represent substrate (34-mer) and product (18-mer) bands, respectively.
(PDF)

Table S1 Oligonucleotides for SIRV2gp26, SIRV2 Hjr, and *S. islandicus* Hje gene synthesis.
(DOC)

Table S2 Oligonucleotides for DNA substrates.
(DOC)

Table S3 Proteins that interact with MBP-SIRV2 Hjr.
(DOC)
