# Supplementary material for: Biochemical Characterization of a Structure-Specific Resolving Enzyme from Sulfolobus islandicus Rod-Shaped Virus 2
Source: PLoS One. 2011 Aug 17;6(8):e23668. doi: 10.1371/journal.pone.0023668 (PMC3157427; doi:10.1371/journal.pone.0023668)
Supplement: Figure S1 — Four-way junction DNA structures and sequences. (A) A poly(AT)20 cassette in pUC(AT) can form a four-way junction structure. (B) pEMM2 contains a four-way junction sequence at the junction of SIRV2 concatamers. These figures represents one of many conformations of possible mobile four-way junction structures. The four-way junction center may shift from what is represented. Gray regions are vector sequence. (PDF) [file pone.0023668.s001.pdf]

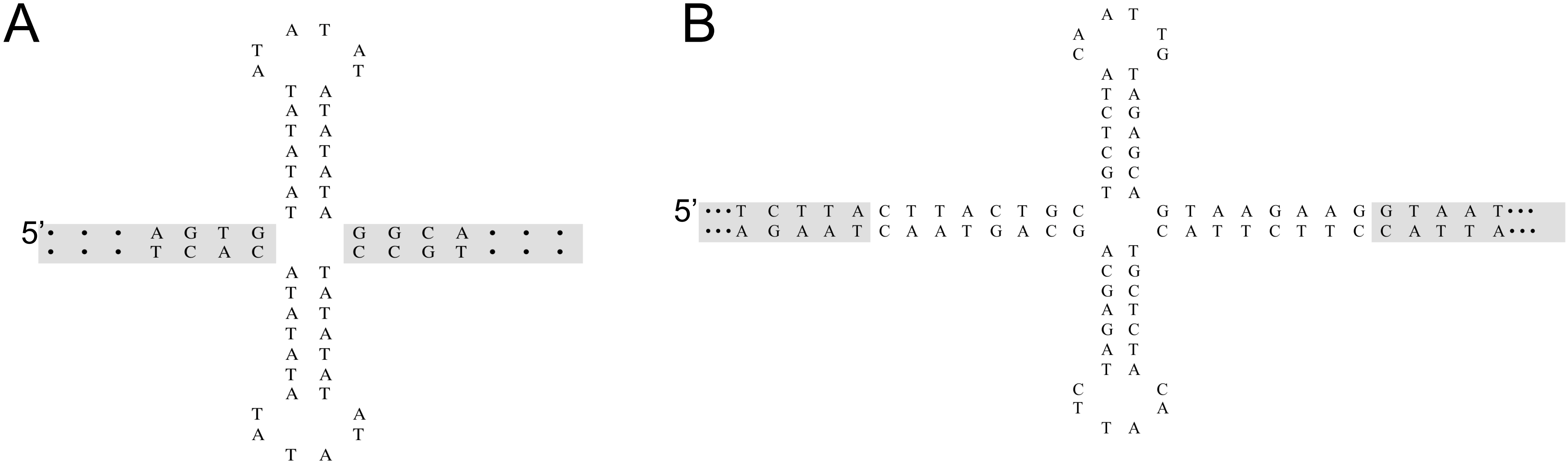

pUC(AT) cruciform

pEMM2 cruciform
