# Supplementary figures and images for: Biochemical Characterization of a Structure-Specific Resolving Enzyme from Sulfolobus islandicus Rod-Shaped Virus 2
Source: PLoS One. 2011 Aug 17;6(8):e23668. doi: 10.1371/journal.pone.0023668 (PMC3157427; doi:10.1371/journal.pone.0023668)

A

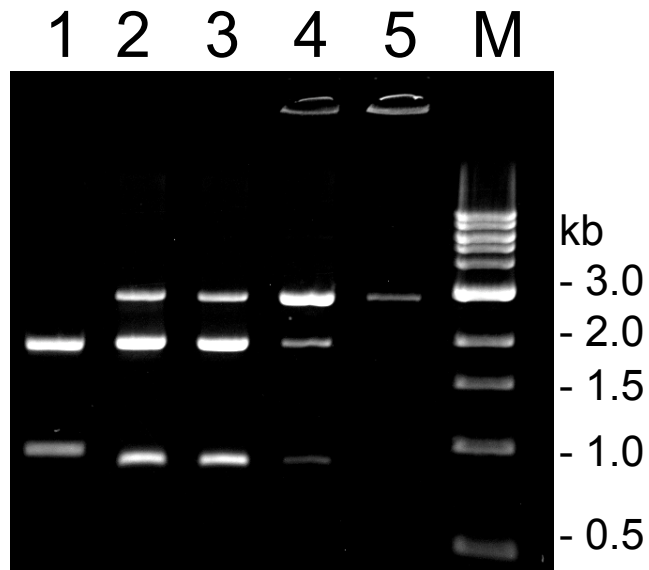

B

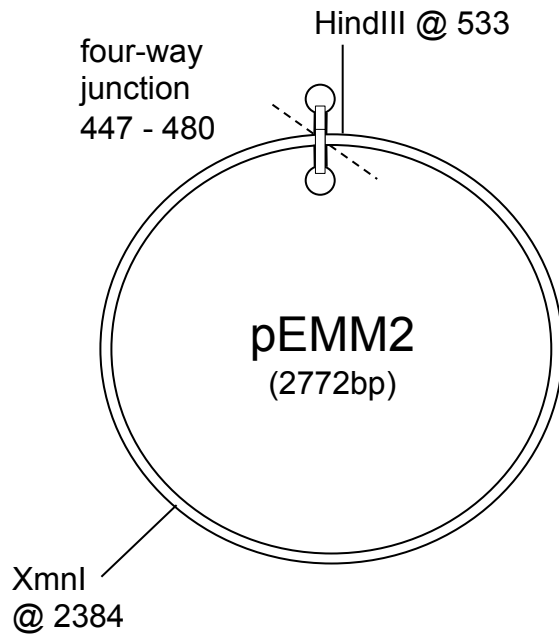

Supplement: Figure S2 — Mapping resolvase cleavage site on plasmid pEMM2. Plasmid pEMM2 contains a four-way junction sequence mimicking the four-way junction formed at SIRV2 concatamer junctions. pEMM2 (100 nM) was incubated with T7 endonuclease I (7.5 nM) (Lane 2), MBP-Hjr (20 nM) (Lane 3), or MBP-Hjr (2 nM) (Lane 4) at 37°C (T7 endonuclease I) or 55°C (MBP-Hjr) for 30 minutes. Then reaction products were then digested with 10 Units of XmnI at 37°C for one hour and separated by agarose gel electrophoresis. As a control (Lane 1), 1 µg pEMM2 was digested with 10 Units of XmnI and HindIII (proximal to the four-way junction) to generate a 921 bp and 1851 bp fragment. pEMM2 was digested with XmnI alone (Lane 5), XmnI and HindIII (proximal to the four-way junction). Plasmid not cleaved by T7 endonuclease I or SIRV2 Hjr and then cleaved with XmnI will generate a linear 2772 bp band. As expected, a double stranded break generated from T7 endonuclease I and MBP•SIRV2 Hjr cleavage mapped to the cruciform region of pEMM2 to generate a ∼875 bp and 1897 bp fragment (Lanes 2, 3, 4). (B) A plasmid map of pEMM2 illustrating HindIII, XmnI and cruciform cleavage site. (PDF) [file pone.0023668.s002.pdf]

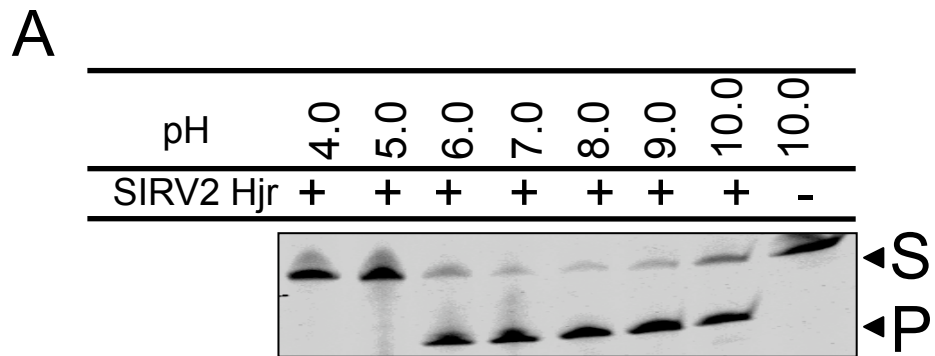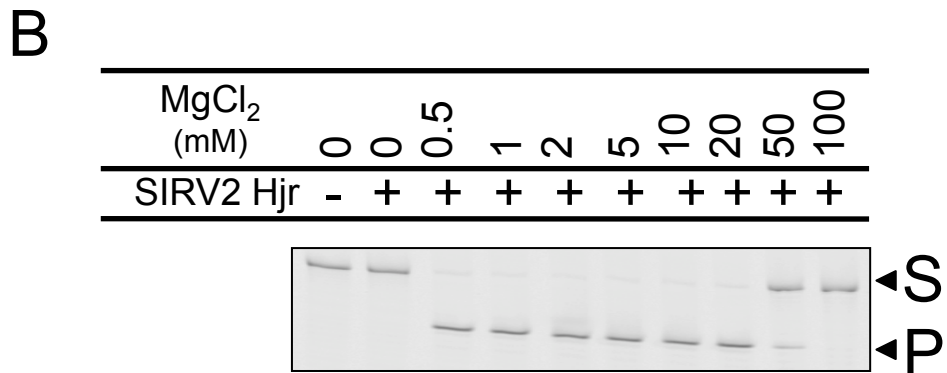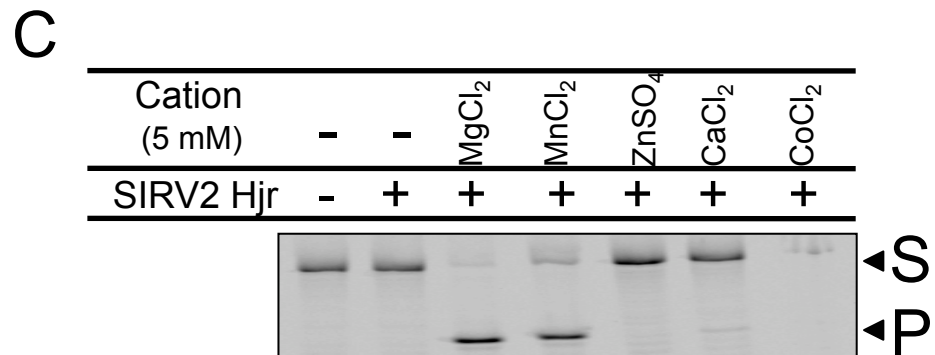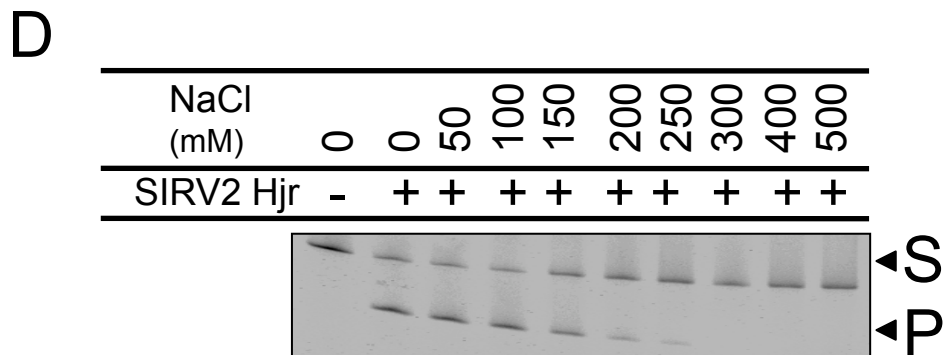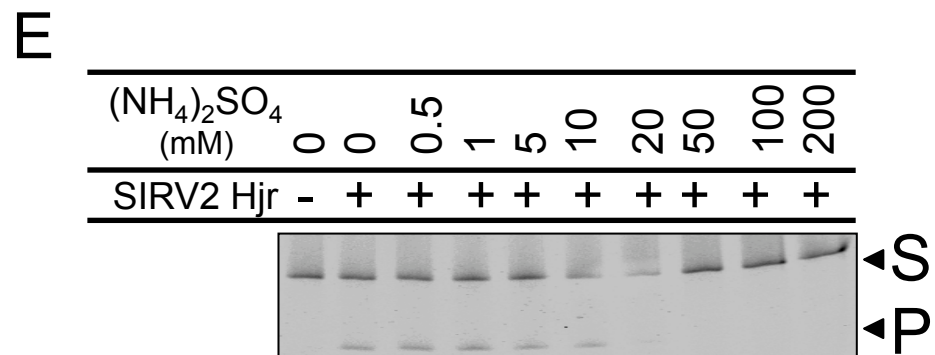

Supplement: Figure S3 — Requirements for SIRV2 Hjr four-way junction cleavage. Hjr cleavage activity was assayed with DNA four-way junction J3 under a variety of reaction conditions to determine requirements for cleavage. Four-way junction J3 (FAM-labeled on strand b) (100 nM) and Hjr (20 nM) were incubated in reaction buffer at 55°C for 30 minutes. Reaction buffers varied pH (4–10), divalent cation (MgCl2, MnCl2, ZnSO4, CaCl2, CoCl2), concentration of NaCl (0–500 mM), NH4SO4 (0–200 mM), or MgCl2 (0–100 mM). Reactions were quenched by addition of 50% formamide and 5 mM EDTA. Reaction products were separated by 20% denaturing PAGE and fluorescence quantified using a GE Typhoon scanner. S and P represent substrate (34-mer) and product (18-mer) bands, respectively. (PDF) [file pone.0023668.s003.pdf]
