# Supplementary material for: Biochemical Characterization of a Structure-Specific Resolving Enzyme from Sulfolobus islandicus Rod-Shaped Virus 2
Source: PLoS One. 2011 Aug 17;6(8):e23668. doi: 10.1371/journal.pone.0023668 (PMC3157427; doi:10.1371/journal.pone.0023668)
Supplement: Table S1 — Oligonucleotides for SIRV2gp26, SIRV2 Hjr, and S. islandicus Hje gene synthesis. (DOC) [file pone.0023668.s004.doc]

Table S1. Oligonucleotides for SIRV2gp26, SIRV2 Hjr, and *S. islandicus* Hje gene synthesis

| Oligonucleotides for SIRV2gp26 gene synthesis | |
| --- | --- |
|  | Oligonucleotide sequence |
| 1 | ATGGCCAAGGGCCATACTAGCCGTTCCT |
| 2 | TGAATTTGGCCTGCCACTTCGCGTAACGCTGAGAGTAGGAACGGCTAGTATGGC |
| 3 | GGCAGGCCAAATTCAATGCTTTTAGCAATCCTACGGTTGCAAGCACCATCC |
| 4 | GAAAATTCTGCTGAGCAACCGGGCTTACATTGGACAGGATGGTGCTTGCAAC |
| 5 | GTTGCTCAGCAGAATTTTCAGACCAACGTCCCGAAGTTCACCAGCGTCAACGAAA |
| 6 | CGGTAATACCGTACTGAGTCAGCACAGCGGATACATTTTCGTTGACGCTGGT |
| 7 | TGACTCAGTACGGTATTACCGGTCCGAACCGCGCAATTTACCAAGGTTTCGGTCTGA |
| 8 | GACCAGAACCGATGCGGTTCAGAGCACGAGCAACTTTCAGACCGAAACCTTGG |
| 9 | CGCATCGGTTCTGGTCCTGCTCTGGTTAACATGATTAACGGTCTGAAAGGCT |
| 10 | GAACCTGCGGATTCGCGTTGAAGGCGCTGATATAATAGCCTTTCAGACCGTTAATC |
| 11 | CGAATCCGCAGGTTCTGGATGCTGTCGTCAACATTATCACCGGCTCCCCAA |
| 12 | TTAGGAAACGTAACCGGTTGGGGAGCCGG |
| Oligonucleotides for SIRV2 Hjr gene synthesis | |
|  | Oligonucleotide sequence |
| 1 | ATGAACATTCGTCAGTCTGGTAAATATTACGAATAT |
| 2 | GCTTTGAAACCGTTTTCCTCCAGGATCTCCAGGGTTTTATATTCGTAATATTTACCAGACTGACG |
| 3 | GGAAAACGGTTTCAAAGCCCTGCGTATTCCGGTTAGCGGTACTGGTAAGCAGGCGC |
| 4 | GTAGATGGTGTTGTCTTTGGTAGCAATGATATCCGGCAGCGCCTGCTTACCAG |
| 5 | CTACCAAAGACAACACCATCTACCCGATTGAAGTGAAGTCTACCAGCAAGGATGTTATCAC |
| 6 | AGAATTCGAACAGCTTTTCGATTTGAAATTTTTTGATGGTGATAACATCCTTGCTGGT |
| 7 | CGAAAAGCTGTTCGAATTCTGCGAAATCTTCGACTTCTGCAACTGCCAGCCGCTGGTA |
| 8 | TAAACGATAACGGTCTTGTATTTCTTGTAGTGAATTGCTACCAGCGGCTGGC |
| 9 | GAAATACAAGACCGTTATCGTTTATACTCTGCCGCAAGACGTGCGCGCTAAAGAAAAGATTA |
| 10 | TTAGCTGTTGATGCCGTATTTGAATTTAATCTTTTCTTTAGCGCG |
| Oligonucleotides for *S. islandicus* Hje gene synthesis | |
| 1 | ATGAACCGTGATATCGGTAAGAATGCGGAA |
| 2 | GAAGCCCTCACCGCGCAGGATGGACACCAGTTCACGTTCCGCATTCTTACCGATATC |
| 3 | CGCGGTGAGGGCTTCAACGCAGTACGCATTCCTACCAGCAACTCTTCTCCG |
| 4 | GTTTTCTTTGGTGGCGAAGATATCAGGCAGAGGGTTCGGAGAAGAGTTGCTGGTAG |
| 5 | CTTCGCCACCAAAGAAAACACCCTGCTGTCCATCGAGTGCAAATCCACTTGGGAA |
| 6 | TTTGCGAACCTGGTTTTCTTTCACCTTAACTTTGTTTTCCCAAGTGGATTTGCA |
| 7 | AGAAAACCAGGTTCGCAAACTGTTCGAGTTCCTGTCCATGTTCACCATGTCTGGT |
| 8 | GTGAATCTGTTTGAATTTAACCGCGATAATCGGGATACCAGACATGGTGAACATGG |
| 9 | CGGTTAAATTCAAACAGATTCACGAGTGGCGTGTTCTGATCCCTAAGAAAGCGGAGGAC |
| 10 | TTCGATGCTAATAGTGTTATCGATGGTCACAACGATGTCCTCCGCTTTCTTAGGG |
| 11 | CATCGATAACACTATTAGCATCGAAGACCTGTTTAAAATCCTGGAAAAGTCTGTTGAAGAA |
| 12 | TTACGGGGTCAGGATTTTTTCTTCAACAGACTTTTCCAGG |
