# Supplementary material for: Biochemical Characterization of a Structure-Specific Resolving Enzyme from Sulfolobus islandicus Rod-Shaped Virus 2
Source: PLoS One. 2011 Aug 17;6(8):e23668. doi: 10.1371/journal.pone.0023668 (PMC3157427; doi:10.1371/journal.pone.0023668)
Supplement: Table S2 — Oligonucleotides for DNA substrates. (DOC) [file pone.0023668.s005.doc]

Table S2. Oligonucleotides for DNA substrates

| Name | Oligonucleotide sequence |
| --- | --- |
| Bulge top | FAM-CGCCAGGGTTTTCCCAGTCACGAC |
| Bulge bottom | GTCGTGACTGGGAAGGCCCTGGCG |
| Junction 3 b | (FAM)-CCTCCGTCCTAGCAAGGGGCTGCTACCGGAAGGG |
| Junction 3 h | (FAM)-CCCTTCCGGTAGCAGCCTGAGCGGTGGTTGAAGG |
| Junction 3 r | (FAM)-CCTTCAACCACCGCTCAACTCAACTGCAGTCTGG |
| Junction 3 x | (FAM)-CCAGACTGCAGTTGAGTCCTTGCTAGGACGGAGG |
| Hairpin(H1) | FAM-GATTACCTTCTTACTGCTCTACAATTGTAGAGCAGTAAGAAGGTAATC |
| 16-mer standard | FAM-CCCTTCCGGTAGCAGC |
| 18-mer standard | FAM-CCCTTCCGGTAGCAGCCT |
| dsDNA template | AACCGGTTACGTACGTACGTGTCGTGACTGGGAAAACCCTGGCG |
