# Supplementary material for: Biochemical Characterization of a Structure-Specific Resolving Enzyme from Sulfolobus islandicus Rod-Shaped Virus 2
Source: PLoS One. 2011 Aug 17;6(8):e23668. doi: 10.1371/journal.pone.0023668 (PMC3157427; doi:10.1371/journal.pone.0023668)
Supplement: Table S3 — Proteins that interact with MBP-SIRV2 Hjr. (DOC) [file pone.0023668.s006.doc]

Table S3. Proteins that interact with MBP-SIRV2 Hjr

| **Data**  **Set** | **Spectra**  **(#)** | **Distinct Peptides** | **MS/MS Score** | **% AA Coverage** | **Accession (#)** | **Annotation** |
| --- | --- | --- | --- | --- | --- | --- |
| Replication & Repair | | | | | | |
| 0308 | 2 | 2 | 23 | 18 | Sis1215 | Alba DNA binding |
| 0208 | 8 | 2 | 27 | 9 | Sis3080 | Sso10a DNA binding protein |
| 0308 | 2 | 2 | 22 | 9 | Sis3080 | Sso10a DNA binding protein |
| 0308 | 1 | 1 | 16 | 6 | Sis2978 | Single-stranded binding protein (ssb) |
| 0208 | 5 | 3 | 53 | 35 | Sis2978 | Single-stranded binding protein (ssb) |
| 0208 | 15 | 3 | 40 | 45 | Sis1229 | Cren7 DNA binding protein |
| 0308 | 3 | 2 | 25 | 34 | Sis1229 | Cren7 DNA binding protein |
| SIRV2 Proteins | | | | | | |
| 0208 | 22 | 4 | 67 | 35 | SIRV2gp35 | SIRV2 Hjr |
| 0308 | 5 | 4 | 60 | 35 | SIRV2gp35 | SIRV2 Hjr |
| 0308 | 2 | 1 | 19 | 22 | SIRV2gp26 | SIRV2gp26  coat protein |
| 0208 | 4 | 1 | 20 | 22 | SIRV2gp26 | SIRV2gp26  coat protein |
| Transcription | | | | | | |
| 0208 | 4 | 1 | 18 | 16 | Sis860 | NusA transcription elongation factor |
| 0208 | 1 | 1 | 17 | 4 | Sis864 | elongation factor 1 alpha |
| Translation | | | | | | |
| 0308 | 1 | 1 | 17 | 8 | Sis792 | Ribosomal protein |
| 0308 | 3 | 3 | 47 | 15 | Sis1052 | 50S ribosomal subunit |
| 0208 | 12 | 4 | 60 | 19 | Sis1052 | 50S ribosomal subunit |
| 0308 | 1 | 1 | 17 | 13 | Sis1051 | 50S ribosomal subunit |
| 0308 | 1 | 1 | 15 | 14 | Sis1038 | 50S ribosomal subunit |
| 0208 | 5 | 2 | 24 | 19 | Sis1038 | 50S ribosomal subunit |
| 0208 | 4 | 1 | 18 | 10 | Sis1037 | 50S ribosomal subunit |
| 0208 | 9 | 3 | 38 | 14 | Sis675 | 30S ribosomal subunit |
| Metabolism | | | | | | |
| 0308 | 5 | 4 | 58 | 18 | Sis1497 | NAD glutamate dehydrogenase |
| 0208 | 24 | 7 | 112 | 29 | Sis1497 | NAD glutamate dehydrogenase |
| 0208 | 2 | 2 | 18 | 16 | Sis1047 | NAD glutamate dehydrogenase |
| 0308 | 2 | 2 | 23 | 4 | Sis1127 | Chaperonin |
| 0208 | 12 | 7 | 93 | 14 | Sis1127 | Chaperonin |
| 0208 | 5 | 3 | 39 | 8 | Sis2333 | Chaperonin |
| 0208 | 16 | 6 | 87 | 16 | Sis805 | Chaperonin |
| 0208 | 1 | 1 | 20 | 6 | Sis116 | Oxidoreductase |
| 0308 | 1 | 1 | 20 | 6 | Sis116 | Oxidoreductase |
| 0208 | 3 | 3 | 34 | 10 | Sis801 | fructose 1,6-bisphosphatase |
| 0208 | 4 | 3 | 30 | 11 | Sis555 | Phosphoglycerate kinase |
| 0208 | 4 | 2 | 27 | 19 | Sis1030 | Adenylate kinase |
| 0208 | 2 | 2 | 23 | 11 | Sis679 | homocysteine methyltransferase |
| 0208 | 2 | 2 | 17 | 22 | Sis1095 | proteasome subunit |
| Hypothetical proteins | | | | | | |
| 0308 | 2 | 2 | 31 | 23 | Sis905 | Zn-finger conserved hypothetical |
| 0208 | 2 | 2 | 31 | 23 | Sis905 | Zn-finger conserved hypothetical |
| 0308 | 2 | 2 | 24 | 18 | Sis503 | Conserved hypothetical protein |
| 0208 | 2 | 2 | 24 | 18 | Sis503 | Conserved hypothetical protein |
| 0208 | 1 | 1 | 18 | 32 | Sis1380 | Conserved hypothetical protein |
| 0208 | 1 | 1 | 18 | 32 | Sis1530 | Conserved hypothetical protein |
